# Large-scale synthesis and luminescence of GdPO_4_ hollow microspheres

**DOI:** 10.1039/c8ra04198a

**Published:** 2018-06-13

**Authors:** Yu Gao, Yongkun Qiu, Xin Wang, Yanfeng Bi, Guiyan Zhao, Fu Ding, Yaguang Sun, Zhenhe Xu

**Affiliations:** College of Chemistry, Chemical Engineering and Environmental Engineering, Liaoning Shihua University Fushun 113001 P. R. China biyanfeng@lnpu.edu.cn; The Key Laboratory of Inorganic Molecule-Based Chemistry of Liaoning Province, College of Applied Chemistry, Shenyang University of Chemical Technology Shenyang 110142 China xuzh@syuct.edu.cn dingfu@syuct.edu.cn

## Abstract

GdPO_4_ hollow microspheres were synthesized by using a novel multi-step transformation synthetic route for the first time with polystyrene (PS) spheres as the template, followed by the combination of a facile homogeneous precipitation method, an ion-exchange process, and a calcination process. The XRD results indicated that the GdPO_4_ hollow microspheres have a pure hexagonal phase. The SEM and TEM images confirmed that the as-obtained GdPO_4_ hollow spheres have a uniform morphology with an average diameter of 2.7 μm and shell thickness of about 150 nm. The up-conversion luminescence properties as well as the emission mechanisms of the GdPO_4_:Yb^3+^, Ln^3+^ (Ln^3+^ = Tm^3+^, Er^3+^ and Ho^3+^) hollow microspheres were systematically investigated, which show blue (Tm^3+^, ^1^G_4_ → ^3^H_6_), green (Er^3+^, ^4^S_3/2_, ^2^H_11/2_ → ^4^I_15/2_), and red (Ho^3+^, ^5^F_5_ → ^5^I_8_) luminescence under 980 nm NIR excitation, providing potential applications in bioanalysis, optoelectronic and nanoscale devices, future color displays and light-emitting devices, and so on.

## Abstract 2

GdPO_4_ hollow microspheres were synthesized by using a novel multi-step route for the first time with polystyrene (PS) spheres as the template, followed by the combination of a facile homogeneous precipitation method, an ion-exchange process, and a calcination process.

## Graphical abstract

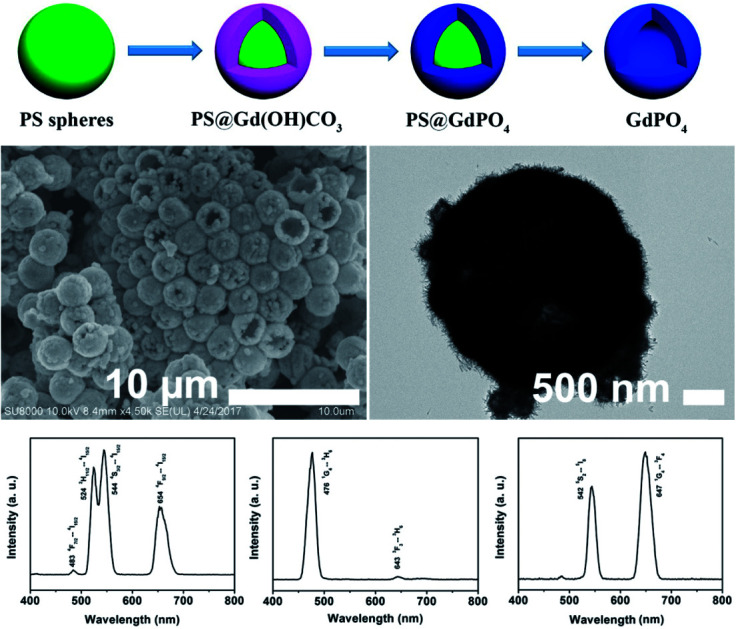

## 1. Introduction

In recent years, inorganic hollow spheres with remarkable interior spaces and shells have attracted interest in modern chemistry and materials research areas owing to their higher specific surface area, lower density and better permeation and widespread potential applications in chemical reactors, sensors, drug delivery, catalysis and various new application fields.^1–7^ With the development of these functional materials, a variety of fabrication methods have been exploited, such as the Kirkendall effect,^8–10^ galvanic replacement,^11,12^ the template route,^4,13^ chemical etching,^14,15^ and thermal decomposition.^16^ Among all these commonly used strategies, on the basis of hard templates (*e.g.*, silica spheres,^17^ carbon spheres,^18^ and melamine formaldehyde^19^) or building on soft templates (*e.g.*, bacteria,^20^ gas bubbles,^21^ and vesicles^22^), the template route is the most usual and efficient way of obtaining hollow structures. Among various hard templates, polymer latex particles, especially polystyrene (PS) spheres, have been reported to be effective templates for the preparation of hollow spherical inorganic materials such as TiO_2_,^23^ SiO_2_,^24^ and Ta_2_O_3_.^25^ Although some progress has been made in the preparation of hollow structures it is still a challenge to develop more facile, efficient, and low-cost techniques to fabricate large-scale and well-crystallized hollow structures.

Recently, rare earth orthophosphates (REPO_4_) have been extensively studied since their potential applications in color displays, field-effect transistors, optoelectronics, solar cells, and light sources.^9,10,26^ Among the different kinds of rare earth orthophosphates, gadolinium orthophosphate (GdPO_4_) is a very important host material for phosphor for Stokes shifted luminescence and has high thermal and chemical stability because Gd^3+^ has a half-filled 4f electron shell with a stable structure.^27^ In particular, compared with conventional down-conversion luminescence materials (organic dyes and semiconductor quantum dots), up-conversion (UC) luminescence has advantages such as narrow emission peaks, long lifetimes, large Stokes shifts, superior photostability, and low toxicity.^3,7,28–30^ Especially, GdPO_4_ is of particular interest for doping rare earth ions as it can act as efficient energy converter from IR to UV and visible emissions. Meanwhile, because of the presence of a large number of unpaired electrons in the gadolinium ion, complex species of Gd^3+^ have been commonly used as MR contrast agents for positive intensity images.^31^ Therefore, much more efforts have been devoted to exploit state-of-the-art methods for the synthesis of GdPO_4_ with different morphologies such as hydrothermal reactions,^10^ co-precipitation route,^32^ pechini type sol–gel,^27^ and solid-state reaction.^27^ However, to the best of our knowledge, the reports on the synthesis of uniform and well-dispersed Ln^3+^-doped GdPO_4_ hollow microspheres using the template method are still limited.

Herein, we report a facile process for the synthesis of uniform GdPO_4_:Yb^3+^/Ln^3+^ (Ln^3+^ = Tm^3+^, Er^3+^, Ho^3+^) hollow microspheres, using urea as a precipitating agent and colloidal PS spheres as templates, followed by hydrothermal and annealing process. The structure, morphology, formation process and up-conversion luminescence properties of the as-obtained GdPO_4_:Yb^3+^/Ln^3+^ (Ln^3+^ = Tm^3+^, Er^3+^, Ho^3+^) hollow microspheres have been well investigated in detail. Moreover, our method is economical, environmentally friendly, and conducive to high-yield mass production, which may pave the way to synthesize hollow spheres of other oxides and extend their applications.

## 2. Experimental section

### 2.1 Materials

The rare-earth oxides Gd_2_O_3_ (99.99%), Yb_2_O_3_ (99.99%), Er_2_O_3_ (99.99%), Tm_2_O_3_ (99.99%), Ho_2_O_3_ (99.99%) and other chemicals were purchased from Aladdin Reagent Co. Ltd. Rare earth chloride stock solutions of 0.2 M were prepared by dissolving the corresponding metal oxide in hydrochloric acid at an elevated temperature. All chemicals were analytical-grade reagents and used as purchased without further purification.

### 2.2 Preparation of monodispersed PS microspheres

In a typical synthesis, the poly(*N*-vinylpyrrolidone) K30 stabilizer (1.0 g) was dissolved in ethanol (38.2 mL) in a three-necked round bottom flask fitted with a condenser and a magnetic stirrer. The reaction vessel was then heated to 70 °C under a nitrogen blanket and purged with nitrogen for 2 h. Then, A solution of azoisobutyronitrile (0.15 g) pre-dissolved in styrene monomer (15 g) was added to the reaction vessel with vigorous stirring. The styrene polymerization was allowed to proceed for 12 h before cooling to room temperature. The product was purified by repeated centrifugation and washed with ethanol. A white fine powder (PS) was finally obtained after being dried in a vacuum oven at 50 °C.

### 2.3 Preparation of the monodisperse GdPO_4_ hollow microspheres

First, 1 mmol of GdCl_3_ aqueous solution and the as-prepared PS microspheres (100 mg) were added to 50 mL deionized water and well dispersed with the assistance of ultrasonication for 30 min. Then, 2.0 g of urea was dissolved in the solution under vigorous stirring. Finally, the mixture was transferred into a 100 mL flask and heated at 90 °C for 2 h with vigorous stirring before the product was collected by centrifugation. The product was washed by deionized water and ethanol three times. Second, the as-obtained sample was dispersed deionized water by ultrasonic for 30 min. Then, 0.2 g of NH_4_H_2_PO_4_ dissolved in an appropriate amount of deionized water was dripped into the dispersion followed by further stirring. After additional agitation for 60 min, the as-obtained mixing solution was transferred into a Teflon bottle held in a stainless steel autoclave, sealed and maintained at 180 °C for 24 h. As the autoclave was cooled to room temperature naturally, the precipitate was separated by centrifugation, washed with deionized water and ethanol in sequence, and then dried in air at 80 °C for 12 h. Finally, the final GdPO_4_ hollow microspheres were obtained through a heat treatment at 800 °C in air for 4 h with a heating rate of 1 °C min^−1^. The GdPO_4_:Yb^3+^/Ln^3+^ (Ln^3+^ = Tm^3+^, Er^3+^, Ho^3+^) hollow microspheres were prepared in a similar procedure except that by adding corresponding YbCl_3_, ErCl_3_, TmCl_3_, and HoCl_3_ together with GdCl_3_ as the starting materials as described above.

### 2.4 Characterization

Powder X-ray diffraction (XRD) measurement was performed on a Rigaku-Dmax 2500 diffractometer with Cu Kα radiation (*λ* = 0.15405 nm). Thermogravimetric analysis (TGA) data was recorded with Thermal Analysis Instrument (SDT 2960, TA Instruments, New Castle, DE) with the heating rate of 10 °C min^−1^ in an air flow of 100 mL min^−1^. The morphologies and composition of the as-prepared samples were inspected on a field emission scanning electron microscope (FE-SEM, SU8010, Hitachi). Low- to high-resolution transmission electron microscopy (TEM) was performed using FEI Tecnai G^2^ S-Twin with a field emission gun operating at 200 kV. Images were acquired digitally on a Gatan multiple CCD camera. The up-conversion emission spectra were obtained using a 980 nm laser from an OPO (optical parametric oscillator, Continuum Surelite, USA) as the excitation source and detected by R955 (HAMAMATSU).

## 3. Results and discussion

**Scheme 1:**
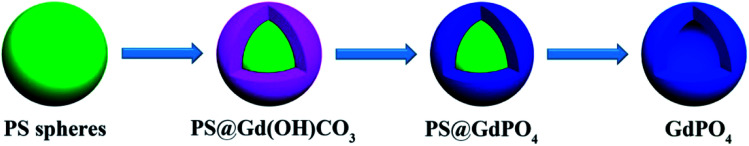
Schematic representation of the synthesis processes of the GdPO_4_ hollow microspheres.

**Fig. 1:**
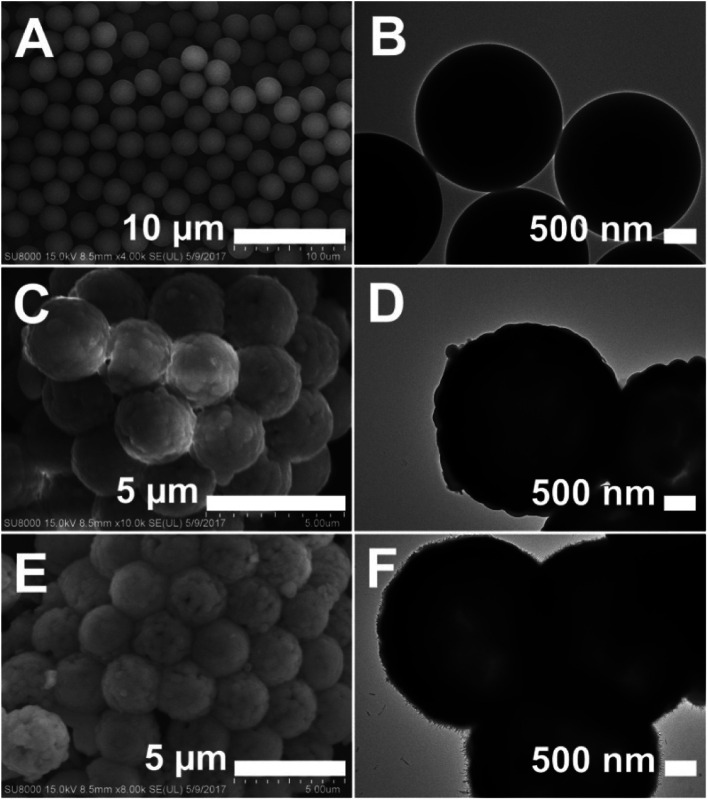
SEM and TEM images of (A and B) the PS spheres, (C and D) the PS@Gd(OH)CO_3_ microspheres, and (E and F) the PS@GdPO_4_ microspheres.

**Fig. 2:**
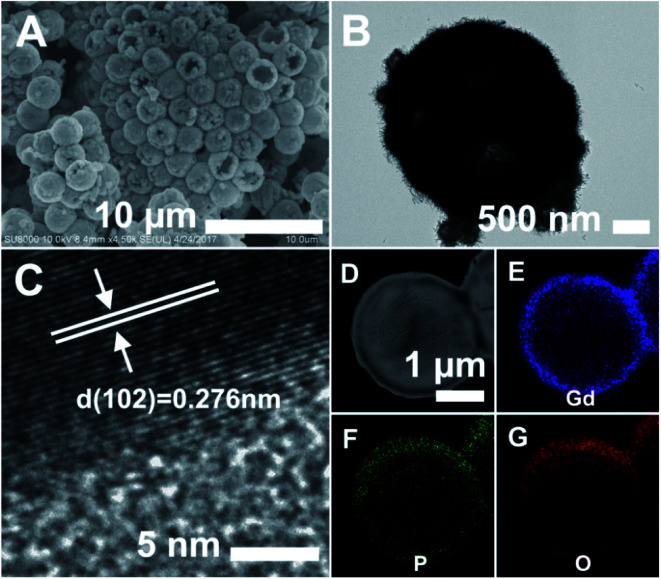
SEM (A), TEM (B), and HRTEM (C) images of the GdPO_4_ hollow microspheres. (D) HAADF-STEM image of the GdPO_4_ hollow microsphere, and the corresponding elemental maps for (E) Gd, (F) P, and (G) O.

Scheme 1 illustrates the synthesis route for the hollow GdPO_4_ microspheres. Monodispersed PS microspheres were chosen as templates, followed by the combination of a facile homogeneous precipitation method, an ion-exchange process, and a calcination process. The morphology evolution from PS microspheres to PS@GdPO_4_ microspheres were monitored as shown in Fig. 1 and 2. The PS microspheres consist of well-dispersed microspheres with an average size of 2.4 μm, and their surfaces are smooth (Fig. 1A and B). After the homogeneous precipitation reaction, Gd(OH)CO_3_ layers were coated around the PS microspheres template (denoted as PS@Gd(OH)CO_3_). From the SEM image (Fig. 1C), it can be seen that the sample inherits the spherical morphology, and the surfaces are much rougher than those of the PS microspheres template because of the precipitation of a large amount of nanoparticles. The size of the PS@Gd(OH)CO_3_ is about 2.8 μm. Fig. 1D presents the typical representative TEM image of the synthesized PS@Gd(OH)CO_3_ sample, which consists of rough surface microspheres and the core–shell structures can be easily found *via* different colors of cores and shells. The average size of the as-prepared sample 2.8 μm in diameter and the thickness of the shell is about 300 nm. So the size of the PS@Gd(OH)CO_3_ microspheres is larger than that of the pure PS microspheres, which further confirm the formation of the Gd(OH)CO_3_ layer. When the PS@Gd(OH)CO_3_ microspheres was treated with NH_4_H_2_PO_4_ under hydrothermal conditions at 180 °C for 24 h, the product (denoted as PS@GdPO_4_) largely inherit the shape and dimension of the PS@Gd(OH)CO_3_ core–shell microspheres (Fig. 1E). The size of the product is similar to the PS@Y(OH)CO_3_ core–shell microspheres in the size range of 2.8 μm. From the TEM image (Fig. 1F), it can be seen that the average size of the core–shell microspheres is about 2.8 μm and the shell thickness is about 300 nm, which conforms to the size calculated from the SEM image.

Finally, the PS@GdPO_4_ microspheres were calcined at 800 °C in air, realizing the successful formation of the hollow GdPO_4_ microspheres. Fig. 2A and B show the SEM and TEM images of the GdPO_4_ sample, indicating the well-defined hollow structure. The diameter of the hollow GdPO_4_ microspheres is similar to the size of the PS template (2.7 μm in diameter) and the shell thickness is 150 nm. Fig. 2C gives the HRTEM image of the hollow GdPO_4_ microspheres. The obvious lattice fringes confirm the high crystallinity, and the interplanar distance between the adjacent lattice fringes is 0.276 nm. This plane can be indexed as the *d* spacing of the (102) plane of the GdPO_4_ crystal. The high-angle annular dark-field scanning transmission electron microscope (HAADF-STEM) image in Fig. 2D further prove the hollow interior. The energy X-ray spectroscopic (EDS) mapping of Gd, P, and O elements within a single particle is shown in Fig. 2E–G, indicating their homogeneous distribution in the shells.

**Fig. 3:**
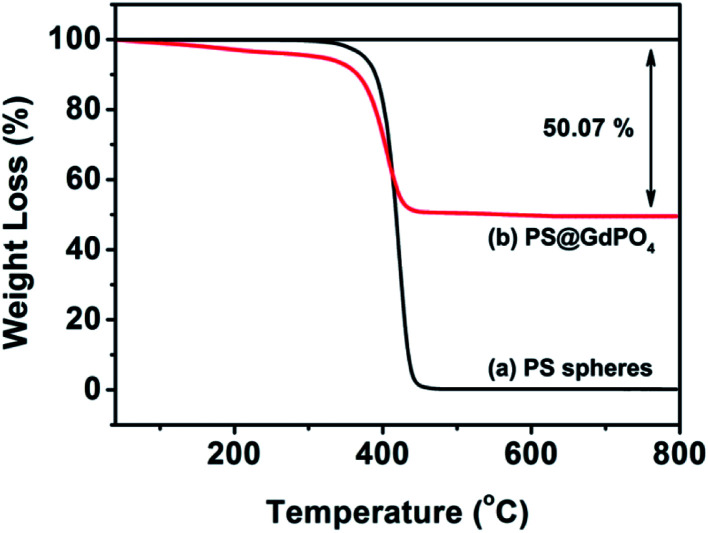
TGA curves of the PS spheres, and the PS@GdPO_4_ microspheres.

The TG analysis as shown in Fig. 3 can give more information about the transformation from the PS@GdPO_4_ microspheres into the hollow GdPO_4_ microspheres during calcination process. The PS microspheres can be completely degraded below 450 °C under air atmospheres. The PS microspheres in the PS@GdPO_4_ microspheres are also completely degraded below 450 °C under air atmospheres, and crystallinity of the final product is also increased. The residual weight percentage is about 49.93%, which accounts for the final GdPO_4_ hollow microspheres, suggesting the considerably high yield of the hollow phosphors prepared using this method. So it can be concluded that the calcination process has a dual function: elimination of the PS sphere cores to form hollow microspheres and the increase of crystallinity of the final product.

**Fig. 4:**
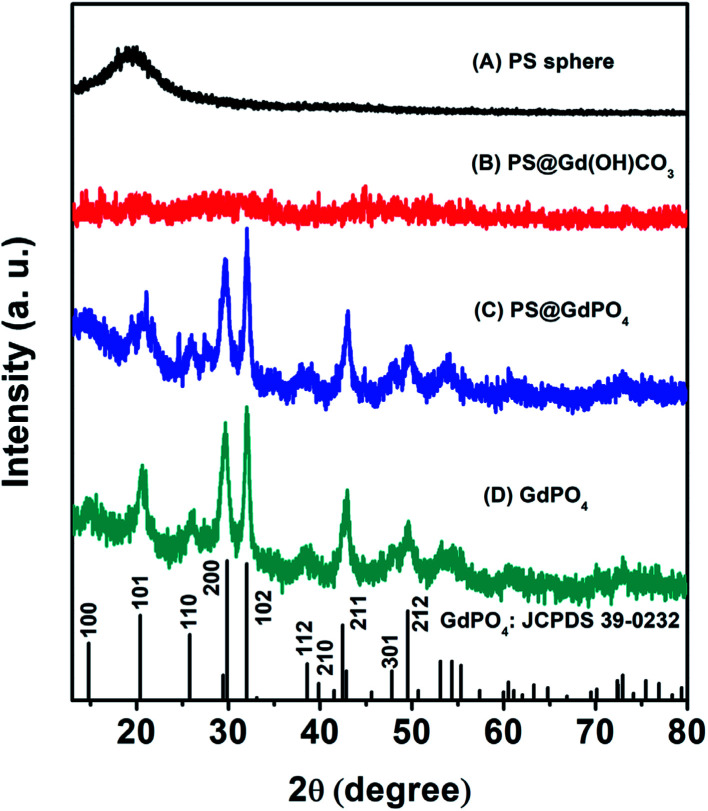
XRD patterns of (A) the PS spheres, (B) the core–shell PS@Gd(OH)CO_3_ microspheres, (C) the core–shell PS@GdPO_4_ microspheres, and (D) the GdPO_4_ hollow microspheres.

The crystal and phase structures of the PS microspheres, the PS@Gd(OH)CO_3_ microspheres, the PS@GdPO_4_ microspheres, and the hollow GdPO_4_ microspheres were then investigated by XRD measurements (Fig. 4). It can be seen that the PS microspheres show an obviously broadened diffraction peak at 19°, which is a typical XRD pattern of the PS spheres. The PS@Gd(OH)CO_3_ microspheres don't show no obvious diffraction peak, indicating that the sample is amorphous. After the PS@Gd(OH)CO_3_ sample was reacted with NH_4_H_2_PO_4_ in the hydrothermal process, all diffraction peaks could be indexed easily as the hexagonal phase of GdPO_4_, in good agreement with the values in the standard cards JCPDS no. 39-0232 for GdPO_4_. Furthermore, the intensity and sharpness of the peaks corresponding to anatase GdPO_4_ were considerably strengthened after calcination, indicating improved crystallinity of GdPO_4_. This is important for phosphors because high crystallinity generally means fewer traps and stronger luminescence.

**Fig. 5:**
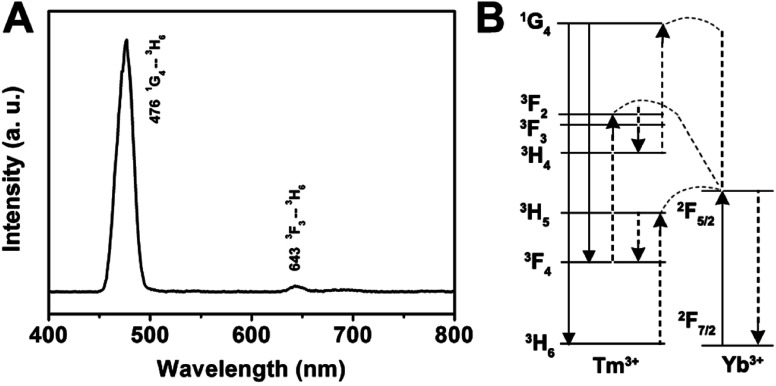
Room-temperature up-conversion spectrum (A) and proposed energy transfer mechanisms (B) of GdPO_4_:Yb^3+^, Tm^3+^.

Herein, we prepared the GdPO_4_:Yb^3+^, Ln^3+^ (Ln^3+^ = Tm^3+^, Er^3+^, Ho^3+^) samples to discuss the up-conversion luminescence properties. All Ln^3+^ doped GdPO_4_ hollow microspheres could be prepared in a similar procedure as for synthesizing GdPO_4_, and the doping concentration of Yb^3+^/Tm^3+^, Yb^3+^/Er^3+^, Yb^3+^/Ho^3+^ was 20 mol%/1 mol%, 20 mol%/1 mol%, and 20 mol%/1 mol%, respectively. Fig. 5A shows the emission spectrum of the GdPO_4_:Yb^3+^, Tm^3+^ sample. The emission bands located at 476 and 643 nm can be attributed to the ^1^G_4_ → ^3^H_6_ and ^3^F_3_ → ^3^H_6_ transition of Tm^3+^, respectively.^33^ The predominant ^1^G_4_ → ^3^H_6_ results in a strong blue emission and ^3^F_3_ → ^3^H_6_ yields a weak red mission. The proposed up-conversion mechanism in GdPO_4_:Yb^3+^, Tm^3+^ is shown in Fig. 5B. The first photon of infrared irradiation elevates an electron to ^2^F_5/2_ and transfer the energy to Tm^3+^, then it can promote an electron from ^3^H_6_ to ^3^H_5_, and Tm^3+^ ion at ^3^H_5_ relaxes non-radiatively to ^3^F_4_. The Tm^3+^ ions in the ^3^F_4_ excited states also can absorb the energy from another Yb^3+^ ion, leading to the Tm^3+^ ion at ^3^F_4_ transits to ^3^F_2_, then decays to ^3^F_3_ and decays non-radiatively from the ^3^F_3_ to ^3^H_6_ with a red emission at 643 nm. At the same time, some Tm^3+^ ions decay from ^3^F_2_ to ^3^H_4_ state, which can be excited by a subsequent Yb^3+^ ion, the Tm^3+^ ion at ^3^H_4_ is excited to ^1^G_4_, and then finally decays non-radiatively to the ^3^H_6_ and ^3^F_4_, producing blue emission at a weak red emission at 476 and 643 nm, respectively.

**Fig. 6:**
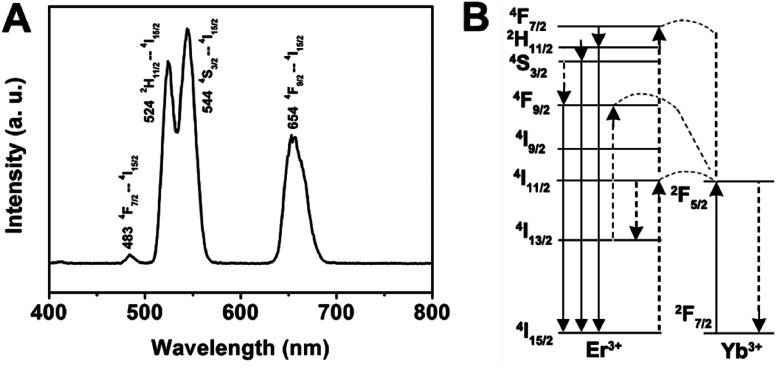
Room-temperature up-conversion spectrum (A) and proposed energy transfer mechanisms (B) of GdPO_4_:Yb^3+^, Er^3+^.

In the case of the GdPO_4_:Yb^3+^, Er^3+^, the sample shows two bands in the green emission region maximized at 524 and 544 nm that are assigned to the ^2^H_11/2_ → ^4^I_15/2_ and ^4^S_3/2_ → ^4^I_15/2_ transitions of Er^3+^, respectively, and a band that appears at about 654 nm is attributed to the ^4^F_9/2_ → ^4^I_15/2_ transition of Er^3+^ (Fig. 6A). The corresponding UC mechanism in the GdPO_4_:Yb^3+^, Er^3+^ sample is described in the energy diagram, as shown in Fig. 6B. The excitation signal (980 nm) is initially absorbed by Yb^3+^ ions to raise the ^2^F_7/2_ to the ^2^F_5/2_ excited state. The ^4^I_11/2_ energy level of the Er^3+^ ions is excited by an initial energy transfer from Yb^3+^ ions in the ^2^F_5_ state. Meanwhile, some of the excited Er^3+^ ions relax rapidly to the low-lying levels of the ^4^I_13/2_ states. Once these states are populated, a subsequent 980 nm photon transferred from the excited-state Yb^3+^ ions can populate a higher ^4^F_7/2_ energetic state of the Er^3+^ ions. The Er^3+^ ions can then decay non-radiatively to the low-lying ^2^H_11/2_ and ^4^S_3/2_ states of the Er^3+^ ions, which result in the dominant green ^2^H_11/2_ → ^4^I_15/2_ and ^4^S_3/2_ → ^4^I_15/2_ emission or further relax and populate a red ^4^F_9/2_ → ^4^I_15/2_ emission.

**Fig. 7:**
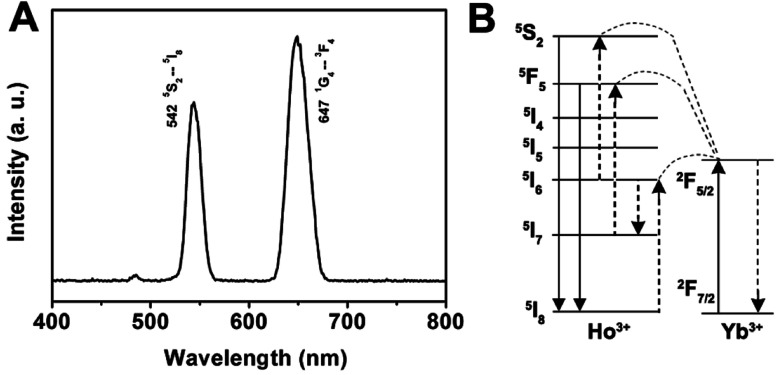
Room-temperature up-conversion spectrum (A) and proposed energy transfer mechanisms (B) of GdPO_4_:Yb^3+^, Ho^3+^.

As for the emission spectrum of the GdPO_4_:Yb^3+^, Ho^3+^ sample in Fig. 7A, the peak centered at 542 nm can be assigned to the ^5^S_2_ → ^5^I_8_ transition, and the peak at 647 nm may originate from the ^5^F_5_ → ^5^I_8_ transition. A possible up-conversion mechanism scheme of GdPO_4_:Yb^3+^, Ho^3+^ is shown in Fig. 7B. It is well known that Ho^3+^ ion does not have an energy level resonant with 980 nm while Yb^3+^ ion has a large absorption band around 1000 nm that matches with the wavelength of 980 nm diode laser very well. Therefore, the energy levels of Ho^3+^ ion are also excited by an initial energy transfer from the excited-state Yb^3+^ ions, then a few subsequent energy transfer processes from Yb^3+^ ions populate the upper Ho^3+^ levels, resulting in the various colors of green emission at 542 nm and a weak red emission at 647 nm, which are ascribed to the ^5^S_2_ → ^5^I_8_ and ^5^F_5_ → ^5^I_8_ transitions of Ho^3+^, respectively.

## 4. Conclusion

In summary, the GdPO_4_ hollow microspheres were synthesized by choosing polystyrene spheres as templates, followed by the combination of a facile homogeneous precipitation approach, an ion-exchange process, and a calcination process. The morphology, crystal structure, elemental analysis, and up-conversion luminescence properties of the as-obtained hollow microspheres were characterized by XRD, TGA, SEM, TEM, and PL, respectively. Upon 980 nm NIR laser excitation, GdPO_4_:Yb^3+^, Ln^3+^ (Ln^3+^ = Tm^3+^, Er^3+^, Ho^3+^) samples exhibits strong blue, green and red up-conversion luminescence, respectively, which may have potential applications in the fields of bioanalysis, optoelectronic and nanoscale devices, future color displays and light-emitting devices, and so on. Moreover, this general, facile synthesis strategy may be extended to the preparation of other inorganic functional materials with hollow spherical morphology.

## Conflicts of interest

There are no conflicts to declare.

## Supplementary Material
